# Negligible risk of zoonotic anisakid nematodes in farmed fish from European mariculture, 2016 to 2018

**DOI:** 10.2807/1560-7917.ES.2021.26.2.1900717

**Published:** 2021-01-14

**Authors:** Maria Letizia Fioravanti, Andrea Gustinelli, George Rigos, Kurt Buchmann, Monica Caffara, Santiago Pascual, Miguel Ángel Pardo

**Affiliations:** 1 Department of Veterinary Medical Sciences, Alma Mater Studiorum University of Bologna, Bologna, Italy; 2 Institute of Marine Biology, Biotechnology and Aquaculture, Hellenic Centre for Marine Research, Attika, Greece; 3 Parasitology and Aquatic Pathobiology, Faculty of Health and Medical Sciences, University of Copenhagen, Copenhagen, Denmark; 4 Instituto de Investigaciones Marinas, CSIC, Vigo, Spain; 5 AZTI, Food Research, Basque Research and Technology Alliance (BRTA), Bizkaia, Spain

**Keywords:** European aquaculture, fish-borne zoonoses, *Anisakis*, risk assessment, European seabass *Dicentrarchus labrax*, gilthead seabream *Sparus aurata*, turbot *Scophthalmus maximus*, rainbow trout *Oncorhynchus mykiss*, Atlantic salmon *Salmo salar*

## Abstract

**Background:**

The increasing demand for raw or undercooked fish products, supplied by both aquaculture and fisheries, raises concerns about the transmission risk to humans of zoonotic fish parasites. This has led to the current European Union (EU) Regulation No 1276/2011 amending Annex III of Regulation (EC) No 853/2004 and mandating a freezing treatment of such products. Zoonotic parasites, particularly anisakid larvae, have been well documented in wild fish. Data on their presence in European aquaculture products, however, are still scarce, except for Atlantic salmon (*Salmo salar*), where the zoonotic risk was assessed as negligible, exempting it from freezing treatment.

**Aim:**

To evaluate the zoonotic Anisakidae parasite risk in European farmed marine fish other than Atlantic salmon.

**Methods:**

From 2016 to 2018 an observational parasitological survey was undertaken on 6,549 farmed fish including 2,753 gilthead seabream (*Sparus aurata*), 2,761 European seabass (*Dicentrarchus labrax*) and 1,035 turbot (*Scophthalmus maximus*) from 14 farms in Italy, Spain and Greece. Furthermore, 200 rainbow trout (*Oncorhynchus mykiss*) sea-caged in Denmark, as well as 352 seabream and 290 seabass imported in Italy and Spain from other countries were examined. Fish were subjected to visual inspection and candling. Fresh visceral organs/fillet samples were artificially digested or UV pressed and visually examined for zoonotic anisakid larvae.

**Results:**

No zoonotic parasites were found in any of the fish investigated.

**Conclusions:**

The risk linked to zoonotic Anisakidae in the examined fish species from European mariculture appears negligible. This study laid the groundwork for considerations to amend the current EU regulation.

## Introduction

In the last decade the diffusion of traditional and new eating habits, linked to the consumption of raw or undercooked fish-based preparations, and the increasing demand for fish products in the European Union (EU) market, have potentially put consumers at higher risk of exposure to fish-borne zoonotic parasite. In this context, the scientific community and food safety authorities are called to collect and provide information on the prevalence, abundance and geographical distribution of fish parasites that are transmissible to humans, as well as to elaborate on strategies aimed at preventing and managing the risk of fish-borne parasitic zoonoses [1]. Among these, anisakiasis, which is caused by nematode larvae belonging to *Anisakis* genus in the marine environment, is considered the main threat to human health and, since its first identification in humans in 1960 [2], thousands of related invasive and allergic syndromes have been reported globally [3-5]. This public health issue mainly occurs in countries, Japan above all, where consumption of raw or undercooked fish is part of the traditional cuisine or is encountering an ever-increasing popularity.

Marine anisakid nematodes have a complex life cycle, involving marine mammals and fish-eating birds as definitive hosts, small crustaceans and fish or cephalopods as intermediate and paratenic hosts respectively, with a transmission route strictly linked to the trophic web in the marine environment. So far, the presence of larval stages of zoonotic Anisakidae, mostly *Anisakis*, but also less importantly *Pseudoterranova* and *Contracaecum*, has been widely documented in wild fish populations [4,6]. In farmed fish, however, it is rarely observed, with few reports in different fish species cultured worldwide [7-13]. According to the “*Scientific Opinion on risk assessment of parasites in fishery products*” provided by the European Food Safety Authority (EFSA) in 2010, the risk of the presence of zoonotic helminths should currently only be considered as negligible for farmed Atlantic salmon (*Salmo salar*), based on evidence produced by a number of scientific and technical documents. This crucial aspect was taken into consideration by EU Commission Regulation No 1276/2011 [14], which confirms as mandatory a freezing treatment of fish products intended to be eaten raw or undercooked but exempts farmed Atlantic salmon from this treatment. Monitoring data concerning other farmed marine fish species in Europe are insufficient. To assess the possible zoonotic risks linked to the consumption of such fish, EFSA (2010) deemed extensive epidemiological surveys necessary.

In the framework of the EU funded H2020 ParaFishControl project “*Advanced Tools and Research Strategies for Parasite Control in European farmed fish*” (www.parafishcontrol.eu), a wide observational survey focused on the presence of Anisakidae zoonotic parasites was carried out in the most farmed marine fish species other than Atlantic salmon in European countries. These species included gilthead seabream (*Sparus aurata*), European seabass (*Dicentrarchus labrax*), turbot (*Scophthalmus maximus*) and marine rainbow trout (*Oncorhynchus mykiss*). The goal was to retrieve data allowing to assess the health hazard to humans caused by Anisakidae zoonotic parasites in these fish.

## Methods

### Fish and fish products sampling

From March 2016 to November 2018, a total of 6,549 farmed marine fish among the most-produced species in the EU were sampled and examined, including 2,753 gilthead seabream, 2,761 European seabass and 1,035 turbot. These three fish species represent 95% of the EU mariculture production excluding Atlantic salmon and they are farmed almost entirely in 19 Mediterranean countries, among which Greece, Spain and Italy are the most important EU producers. In addition 200 marine rainbow trout were also examined. In the EU, production in 2019 amounted to 103,197 tons and 100,476 tons for European seabass and gilthead seabream respectively and 11,423 tons for turbot [15].

Gilthead seabream and European seabass samples were collected from 10 Mediterranean farms: five in Italy (four sea cage farms and one inland farm located in the Tyrrhenian and Adriatic Seas), three in Greece (sea cage farms, two in the Aegean and one in the Ionian Sea), and two in Spain (sea cage farms), while turbot samples were collected from four inland farms located on the Atlantic coast of Spain. Fish from the selected farms were fed with commercial extruded pellets. When feasible, the samples included market size fish as well as categories considered at risk, such as runts, or fish farmed in areas where life cycles of heteroxenous zoonotic parasites could be realised.

A random polietapic and stratified sampling plan with a confidence level of 99% and a margin of error (MoE) of 4–8% was designed [16]. First, according to the data provided by the aquaculture industry in each country, fish species were selected according to their commercial significance (i.e. their proportion (%) in relation with the total aquaculture production) in the EU fish farming production systems. Then, for each selected species some fish farms were chosen according to farming practice characteristics and/or their locations in particular environments, as both of these criteria could be potentially associated with a particular hazard exposure risk. Finally, fish samples within each stratum (fish species/farm) were quarterly broken down into different seasonal subgroups to assure that the entire fish production and the practices of the farms were covered throughout the year and to reach a statistically significant amount of at least 258 fish per farm and a total number of 1,032 fish per species at the end of the survey.

In addition, primary processed products (fresh European seabass and gilthead seabream from farms in Greece, Croatia and Turkey) were collected as whole fish from retail in Italy and Spain. Marine rainbow trout from Denmark were also considered in the study. The reason for these being additionally included was that they are farmed in sea cages, which is considered to constitute a risk for transmission of zoonotic anisakid nematodes [17,18]. Moreover, they represent a peculiar fish product of high commercial value. The Danish maricultured rainbow trout were all sampled at the harvest period (November and December) after they had all been in netpens at sea for 7–8 months (the Danish production period at sea is relatively short and runs from stocking in March–April to harvest and slaughtering in November–December) whereby all examined fish had been exposed maximally to any pathogen in the local environment. Care was taken to collect – from each farm – five of the smallest fish and five of the largest in order to reflect differences in growth and feeding.

Details of amount of examined fish divided by country are indicated in the Table.

**Table:** Number of samples for farmed marine fish collected in several European countries, March 2016–November 2018 (n = 7,391)

Fish species	Number of fish (number of runts included)					
	Italy	Spain	Greece	Denmark	Imported^a^	Total
Turbot	NA	1,035 (0)	NA	NA	NA	1,035 (0)
European seabass	1,571 (520)	65 (65)	1,125 (0)	NA	290 (0)	3,051 (585)
Gilthead seabream	1,563 (520)	65 (65)	1,125 (0)	NA	352 (0)	3,105 (585)
Marine rainbow trout	NA	NA	NA	200 (0)	NA	200 (0)
Total	3,134 (1,040)	1,165 (130)	2,250 (0)	200 (0)	642 (0)	7,391 (1,170)

NA: not applicable.

^a^ Imported fish were sampled from Italian and Spanish markets: 290 European seabass were imported from Greece (n = 215), Turkey (n = 45) and Croatia (n = 30); 352 gilthead seabream were imported from Greece (n = 239), Turkey (n = 52) and Croatia (n = 61).

### Ethical statement

No ethical issues are raised in the present study, since research activities were carried out only on fish sampled on farms during harvesting at market-size, just before the commercialisation and in compliance with the Animal Welfare EU Directive and Recommendations [19,20].

### Procedures for parasite detection

All the fish were weighed, measured and subjected to parasitological examination aimed to detect the presence of anisakid larvae as ‘visible parasites’, according to the current European regulatory framework [21]: each fish was checked by visual inspection of the abdominal cavity and the fillet. The inspection methodologies for the detection of anisakid larvae were optimised following the protocols set up during the EU project Parasite (http://parasite-project.eu/) and shared among the partners involved in the survey.

Furthermore, visceral organs were removed from the body cavity and individually inspected, then chopped. The chopped samples (50 to 200 g) were subjected to artificial digestion using a solution composed by 2 L of tap-water/100 g of tissue, 10 g (1:10,000 NF – United States National Formulary) pepsin powder and 10 mL of 25% HCl (molar concentration: 7.8–7.9 mol/L). The mixture was heated on a magnetic stirrer at 40 ± 2 °C until complete digestion, according to the protocol validated in the EU project Parasite [22]. It was subsequently sieved and visually inspected for the presence of anisakid larvae. Fillets were carefully examined by direct observation, and then cut in four portions by side (right–left) and placed into labelled transparent plastic bags, then pressed to obtain 2 mm thick layers by a hydraulic pressing device. The bags were deep frozen at − 20 °C for at least 24 hours and finally observed under an ultraviolet (UV) light (360 nm) transilluminator in a dark room [23]. For marine rainbow trout, the right fillet of each fish was subjected to artificial digestion while the left fillet examined by the compression method as described above.

The finding of a raphidascarid larva in a fish of one farm during the study, led to further investigation of this farm for this type of parasite which, although not zoonotic and having teleosts as definitive hosts, shows similar transmission pathways to those of Anisakid nematodes. Identification of raphidascarid larvae was carried out at genus level by light microscopy on the basis of morphological features [24] and at the species level by PCR-restriction fragment length polymorphism (RFLP) and sequencing of the internal transcribed spacer (ITS) ribosomal DNA region, following Tedesco et al. (2018) [25].

## Results

All the examined fish, runts included, were negative for the presence of larval stages of zoonotic anisakid nematodes.

The data obtained from this extensive survey show the absence of zoonotic parasites in the fish examined and are comparable to those reported for the farmed Atlantic salmon [26-28], gilthead seabream, European seabass [29], turbot [30] and marine rainbow trout [17,18] in previous studies, leading to consider as negligible the risk of anisakid infection in the most important fish species of European mariculture mentioned above.

One European seabass from an Italian farm, showed the presence of a raphidascarid fourth stage larva encysted on the liver, identified as *Hysterothylacium fabri*. Considering the similarities of the transmission routes with those of anisakids, after this finding, 260 (65 fish/season) European seabass runts from the same farm were further analysed and resulted negative, allowing to speculate that the presence of the *H. fabri* larva was accidental.

## Discussion

The results of this study responded to the recommendation given by EFSA [1] to collect data on the parasite risks in fishery products through wide epidemiological surveys. They allowed to map the ‘*Anisakis* risk’ in European mariculture, defining it as negligible in a representative number of farmed marine fish belonging to the sea-caged rainbow trout, gilthead seabream, European seabass and turbot species.

A recent report in 2016 of two larvae of *A. pegreffii* identified in the visceral organs of one farmed European seabass commercialised in southern Italy [13], confirmed the susceptibility of this species to *Anisakis* spp. infections [31-33]. Unfortunately, no data are available on the farm of origin of the infected European seabass, making it impossible to identify potential risk factors involved in the transmission, as was done for Atlantic salmon [10,11] in Norwegian farms, where runts were found to be infected by nematode larvae of *A. simplex* and *Hysterothylacium aduncum* due to farm management issues.

Considering that transmission of anisakid larvae occurs through the trophic chain [34], the main risk aspects to be monitored are linked to a proper management of the fish farm, primarily focusing on the implementation of correct feeding protocols and appropriate management of fish size classes. In this regard, it should be pointed out that, within the farmed fish population, the runts or ‘loser fish’ generally represent specimens at risk of infection by *Anisakis* or other nematodes with a similar life cycle (e.g. *Hysterothylacium* spp.), because runts are less able to compete for food with bigger fish (harvest quality fish) and so driven to prey to potentially parasitised invertebrates or wild fish that may have entered the cage [10,11].

The findings described in this report represent the current status of marine fish farmed in the EU and highlight the absence of zoonotic anisakids. The approach employed moreover lays the ground for planning surveillance activities in EU fish farming systems, as it appears feasible and reliable for the industry. In this respect, the diagnostic methods in our study could be used as a tool within a Hazard Analysis of Critical Control Point (HACCP)-like system. Such a system would not only have the objective to identify any critical points to be monitored (e.g. introduction of fish only from controlled hatcheries, appropriate feeding strategy, good management practices, etc.) during fish farming, but also aim to obtain a documented parasitological surveillance controlling for the absence of zoonotic parasites along the whole aquaculture production chain over time. This would in turn insure that aquaculture products do not present a health hazard with regard to the presence of zoonotic parasites. A long-lasting application of this internal control system should guarantee an economic return to farmers in terms of better market prices for fish products with a high safety level and a progressive optimisation of surveillance sampling plans with a lower number of fish to be internally examined.

A correct application of a HACCP-like system as internal self-control assessment of critical points linked to the zoonotic risk, was already considered in a previous analysis for the Atlantic salmon [35]. When enhanced with the use of feasible and reliable parasite detection methods for the processed fish products (homogenised fish product, etc.) [36], it might additionally aid to maintain the likelihood of zoonotic parasite occurrence in all farmed fish at a very low level.

To facilitate the application of an internal control system it is essential to have a diagnostic method that is effective, cost-efficient and simple to implement. These requirements seem to be satisfied by the combination of the inspection carried out according to EU regulation, and the UV-light press method used in this and in other recent epidemiological studies. The characteristics of these approaches would allow their application in routine field diagnostics with a great sensitivity improvement.

A limitation of the current study was that, a Fulton’s condition index, a parameter measuring the individual growing pattern of the fish sampled, was not used to define runts. While this might have improved standards, such an index might have been required for numerous other fish, with larger logistical implications. We chose a more practical approach, adapted to commercial fishing constraints, whereby ‘runts’ were selected on site by farmers following the routine selection of harvest quality fish.

## Conclusions

In conclusion, based on the results obtained in this study, the risk of *Anisakis* larvae infection is negligible in fish products deriving from European mariculture activities. Farmed gilthead seabream, European seabass, turbot and marine rainbow trout should therefore be considered suitable, as Atlantic salmon, to benefit from the exemption from freezing treatment provided by EU Regulation No 1276/2011 for fish farming products in the form of “*products intended to be consumed raw, or marinated, salted and any other treated fishery products, if the treatment is insufficient to kill the viable parasite*”.

In association with the implementation of an appropriate voluntary control system at farm level, a long-term epidemiological surveillance will be useful to continuously monitor the risk and ensure high levels of food safety in the European aquaculture products.
